# Trauma-informed psychological support alongside long-acting injectable buprenorphine: clients’ perspectives on the Buvidal psychological support service (BPSS) pilot in Wales

**DOI:** 10.3389/fpsyt.2026.1924312

**Published:** 2026-09-25

**Authors:** Gareth Roderique-Davies, Nyle H. Davies, Darren Quelch, Hannah Jones, Chioma Nwozo, Lucie James, Jan Melichar, James Tucker, Lewis Jones, Emma Dennie, Joshua Noonan, Katy Holloway, Josh Molina, Rosemary Greenwood, Bev John

**Affiliations:** 1 Addictions Research Group, Applied Psychology Research and Innovation Group, Faculty of Life Sciences and Education, University of South Wales, Pontypridd, United Kingdom; 2 Alcohol care and clinical toxicology team, Sandwell and West Birmingham NHS Trust, Birmingham, United Kingdom; 3 Buvidal Psychological Support Service, Newport Road, Cardiff and Vale University Health Board, Cardiff, Wales, United Kingdom; 4 Substance Use Research Group, Crime, Security and Justice Research and Innovation Group, Faculty of Life Sciences and Education, University of South Wales, Pontypridd, United Kingdom

**Keywords:** buprenorphine, Buvidal, long-acting injectable buprenorphine, opioid, psychological intervention, trauma

## Abstract

**Background:**

Opioid use disorder (OUD) is associated with persistent drug use, high relapse rates, and significant morbidity, mortality, and psychosocial harm. While Long-Acting Injectable Buprenorphine (LAIB), marketed as Buvidal, offers improved pharmacological management over traditional opioid substitution therapy (OST), the mental clarity provided by LAIB often surfaces unresolved trauma and mental health needs. Integrating trauma-informed psychological care alongside pharmacological intervention is increasingly recognized as critical to optimize recovery outcomes for individuals with co-occurring psychiatric co-morbidities and substance use disorder.

**Objective:**

This evaluation aimed to explore client experience of the Buvidal Psychological Support Service, a tiered trauma-informed psychological service, piloted to support individuals with OUD receiving LAIB in Wales, UK.

**Methods:**

Qualitative data were derived from nine semi-structured interviews with BPSS service users, exploring their experiences, perceived impacts, and recovery journeys through reflexive thematic analysis.

**Results:**

Findings indicated the transition to LAIB was often transformative but exposed trauma and emotional difficulties previously masked by substance use, making the availability of timely psychological support critical for maintaining abstinence and wellbeing. Clients valued the BPSS’s rapid access, continuity, person-centered care and non-judgmental ethos, reporting enhanced agency, emotional regulation, and social reintegration. Relapse prevention was strongly linked to psychological support alongside pharmacological treatment.

**Conclusion:**

The findings suggest that participants experienced BPSS as a valuable adjunct to LAIB, particularly where increased mental clarity brought trauma-related and emotional difficulties into focus. The pilot highlights the potential value of integrating trauma-informed psychological support within LAIB pathways, while further evaluation is needed to assess transferability and longer-term outcomes.

## 1 Introduction

Characterized by persistent opioid consumption, compulsive drug-seeking *behavior*s, and high relapse rates, Opioid Use Disorder (OUD) is closely linked with increased morbidity, mortality, and substantial social harms (1–3). In the United Kingdom, opioid-related deaths have risen steadily over the past decade highlighting the urgent need for sustainable and effective treatment approaches (4).

Historically, opioid substitution therapy (OST) such as methadone and oral buprenorphine has formed the cornerstone of OUD treatment (5). OSTs demonstrate efficacy in reducing opioid use, mortality, and criminal behavior (6–8). However, these conventional treatments are not free from limitations. Daily supervised dosing regimens often entail a restrictive lifestyle, frequent exposure to stigma, and considerable barriers to consistent engagement (9, 10), factors clients have described as “liquid handcuffs” (11). Such experiences of stigma and control often undermine adherence and the potential for recovery (12).

Amidst these challenges, Long-Acting Injectable Buprenorphine (LAIB, marketed as Buvidal) has emerged as a promising alternative, delivering weekly or monthly sustained-release dosing with the removal of daily pharmacy attendance (5, 13–15).

Buvidal was licensed in the UK in 2018 and recommended by NICE in 2019, followed by the All-Wales Medicines Strategy Group approval later that year. Before the pandemic, use in Wales was limited to small pilot schemes, however the onset of COVID-19 transformed Buvidal from a niche intervention into a strategic public health response with Welsh Government funding a rapid national roll-out to reduce daily supervised OST dosing, limit face-to-face contacts, and maintain OST continuity for a population at high risk of severe infection and transmission. By 2026, Buvidal was established as a core OST option in Wales (16).

Since LAIB is a partial opioid agonist, it produces weaker opioid effects when compared to full agonists like methadone. In addition, LAIB is an antagonist at kappa-opioid receptors. This unique pharmacological profile helps to manage opioid dependence by reducing withdrawal symptoms and cravings while *minimizing* the sedative effects seen with methadone (17–19). As such, patients may experience greater mental clarity using LAIB compared to full agonists like methadone. Initial research evidence indicates increased treatment satisfaction, reductions in opioid consumption, improved quality of life, and decreased stigma, although many patients also experience the resurfacing of unresolved trauma (20–23).

It is increasingly recognized that pharmacological stabilization alone cannot address the full complexity of opioid dependence and UK clinical guidelines recommend integrating psychological support within opioid substitution treatment (OST) services (“Orange Book”, 24). High rates of psychiatric co-morbidities, including anxiety, depression, and post-traumatic stress disorder (25, 26) are common among individuals with OUD and contribute to treatment dropout (27). Further, research supports the link between trauma exposure and substance use maintenance (28–31). Adverse Childhood Experiences (ACEs) play a significant role in OUD, with evidence showing that approximately 40% of individuals with OUD have experienced childhood sexual abuse (32) and that ACEs are a shared risk factor for both mental health and substance use difficulties (33). This trauma exposure increases vulnerability to OUD through mechanisms like heightened sensitivity to opioid reward (34) and self-medication of psychiatric symptoms (30), highlighting the need for trauma-informed psychological support for this population.

However, the evidence base for adjunct psychological interventions remains weak and inconsistent. A Cochrane review of 35 RCTs reported no added benefits of psychological therapies for retention or abstinence in patients receiving OST (35). Later reviews reached similar conclusions, citing high variability across interventions and outcomes (36, 37).

One limitation of these studies is the focus on full agonists like methadone rather than partial agonists such as LAIB. If LAIB is associated with clearer cognitive functioning, this this may create conditions that facilitate engagement with psychological support (16). Previous findings may underestimate psychological therapy benefits when delivered alongside LAIB.

These realities form the rationale for embedding holistic, trauma-informed psychological care alongside pharmacological intervention. The Buvidal Psychological Support Service (BPSS) was established in 2023 as a co-designed 2-year pilot service to better understand and support the psychological needs of those on LAIB in Cardiff and the Vale of Glamorgan, Wales. The BPSS offers tiered, rapid access psychological support, drawing upon trauma-informed principles (38) and evidence-based psychological interventions that are aligned with NICE (2019) guidelines (**Figure 1**). The service’s name was agreed after consultation with service users who all knew LAIB as “Buvidal” (as the only formulation used in Wales) and did not want the word “Trauma” to be in the title.

**Figure 1:**
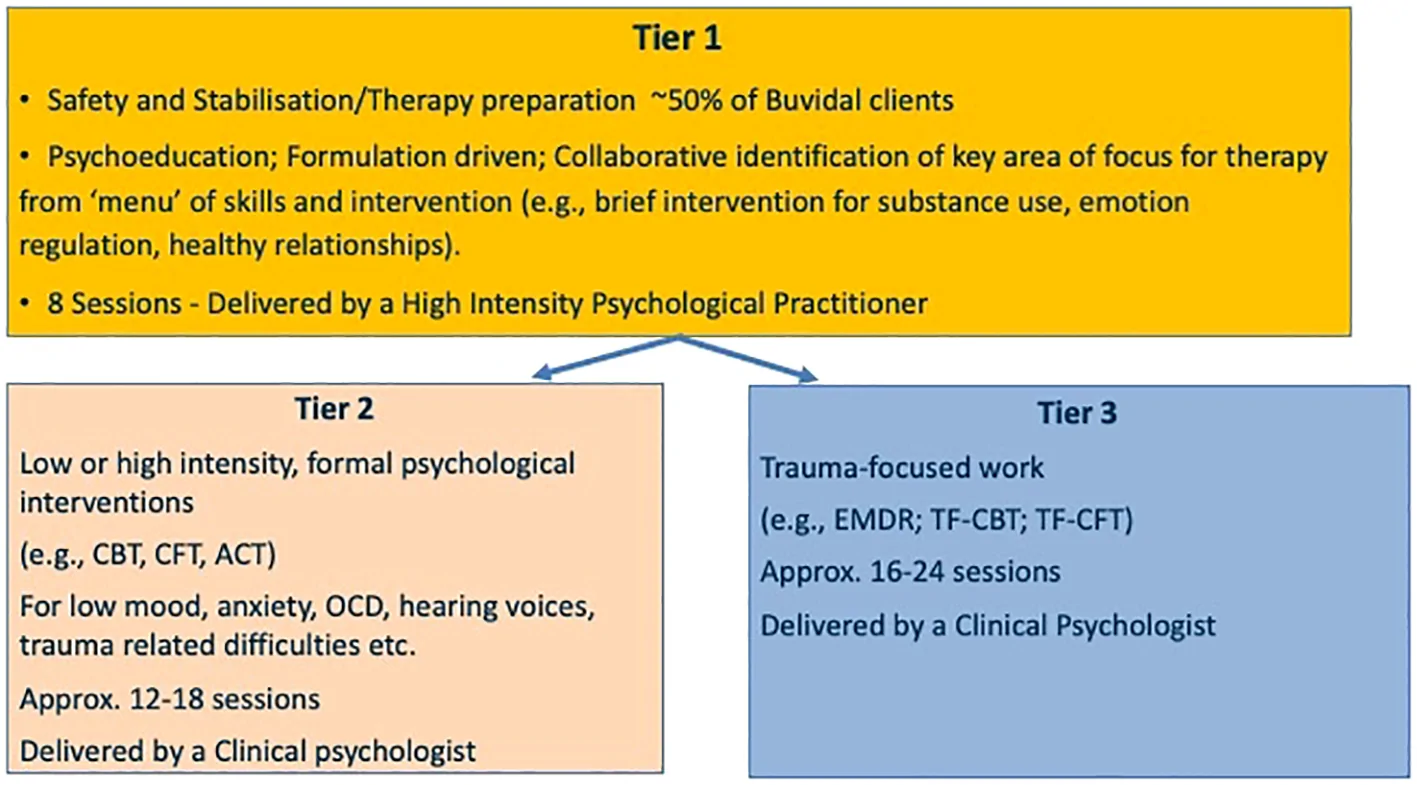
BPSS stepped care model.

Tier 1 offers rapid access to eight weekly sessions of therapy with a qualified therapist. Tier 1 supports clients to make sense of their life experiences through a trauma and compassion-focused lens, addressing the embedded shame and guilt often experienced by this population. Clients are supported to develop skills to address common mental health, addiction and trauma-related difficulties they experience, and to consider ways of reducing maladaptive coping strategies, such as the use of substances to self-medicate. Many clients require no further psychological support; however, some require more intensive psychological interventions to meet their needs through longer-term Tier 2 or 3 specialist intervention. Tier 2 provides up to an additional 18 sessions of formal psychological interventions for trauma-related presentations (e.g. attachment and relational difficulties; emotional dysregulation), addiction and mental health difficulties (e.g., depression, anxiety or hearing voices) with a clinical psychologist. Interventions include Cognitive *Behavioral* Therapy (CBT), Acceptance and Commitment Therapy (ACT), Compassion Focused Therapy (CFT) or an integrated approach. Tier 3 provides up to an additional 24 sessions of targeted evidence-based trauma processing therapy, with a clinical psychologist, based on psychological formulation to tailor intervention to the individual. Interventions (e.g. Eye-Movement *Desensitization* and Reprocessing Therapy (EMDR); Trauma Focused Cognitive *Behavioral* Therapy TF-CBT; Prolonged Exposure (PE)) and may involve integrating a variety of psychological models. Those who are referred to Tier 3 are identified as being able and willing to engage in trauma-focused therapy.

To date, research on LAIB has primarily emphasized pharmacological outcomes—craving suppression, retention, safety—while the lived experiences of clients navigating both LAIB and psychological support remain underexplored (5, 13, 14). Client perspectives are crucial, as satisfaction, engagement, and the extent to which psychological needs are addressed may determine adherence and overall recovery trajectories (21). Moreover, studies to date have not explored LAIB in conjunction with structured psychological support.

The aim of the present study was to explore how people with lived experience of accessing BPSS described the service, and how they perceived its role in supporting recovery alongside LAIB treatment.

## 2 Methods

This study was reported in line with the COREQ guidelines (39).

### 2.1 Ethics

This study was conducted as an independent service evaluation by Cardiff and Vale University Health Board. As such, NHS research ethics committee review was not required; however, relevant Health Board permissions were obtained, and ethical approval for the analysis of the anonymized transcripts was granted by the University of South Wales Faculty of Life Sciences ethics panel. Participants were interviewed about their experiences of the service, data were anonymized prior to analysis, and transcripts were handled and stored securely in line with governance requirements.

### 2.2 Design

Secondary data were obtained from the BPSS, in the form of audio-recordings of semi-structured interviews conducted with clients between March 2024 and June 2024. Six of the interviews (clients P001-P006) were conducted by an Assistant Psychologist, and the remaining three (clients P007-P009) by a Trainee Clinical Psychologist; both were working within the BPSS but had no direct therapeutic involvement with the clients they interviewed. A semi-structured interview topic guide was used to explore participants’ experiences of starting Buvidal, their engagement with BPSS, perceived changes in wellbeing and recovery, and views on what aspects of the service were most helpful or could be improved. The interview guide was co-created with people with lived experience.

### 2.3 Reflexivity

The interviews were conducted by BPSS staff who had no direct therapeutic relationship with the participants interviewed. This positioning offered familiarity with the service context, while also introducing the possibility of socially desirable responding. To mitigate this, transcripts were anonymized and the university evaluation team undertook reflexive thematic analysis collaboratively, with attention to how researcher perspectives and service context may have shaped interpretation.

### 2.4 Participants

All eligible BPSS clients who had completed at least Tier 1 support during the evaluation period were invited to take part through the service, and 9 agreed to participate. Reasons for declining participation weren’t recorded. The participants included five males and four females whose ages ranged from 31 to 55 years. Seven reported heroin as the primary substance leading to their engagement with OST, one reported “other prescription drugs” and one reported “other opiate.”

All the participants accessed “Tier 1” support completing a minimum of 8 one-to-one therapy sessions, with two participants also accessing “Tier 2” and one participant accessing “Tier 3”. None of the participants accessed all 3 Tiers. Participant ID codes were assigned by the University evaluation team and are not related to any identification codes used by BPSS.

### 2.5 Procedure

Interview modalities available to clients were face-to-face, online and telephone. Three clients opted for face-to-face (P005, P008, P009), one online (P001), with the remaining five (P002, P003, P004, P006, P007) taking part by telephone. Interviews were transcribed verbatim using NHS computers at an approved health board site.

### 2.6 Analysis

Reflexive Thematic Analysis (RTA; 40) was used to identify salient patterns across clients’ experiences. Inductive RTA was adopted, meaning that coding was driven by the data. Guided by Braun and Clarke’s contemporary RTA methodology, analysis was conducted by multiple researchers. Data familiarization was achieved independently through readings of all transcripts. This exploratory process was focused on capturing ideas and scope; annotations were made in transcript margins and code labels attached. A complete coding approach was taken, as the researchers sought to identify anything of interest across the entire data set. In-depth readings followed, and initial themes were developed. The University evaluation team then collaborated to reflect on differences in coding and theme generation. This reflects contemporary RTA methodology which embraces researcher subjectivity and prioritizes richer interpretations of meaning over attempts to arrive at consensus between coders (40, 41).

Despite a relatively small sample, the research team’s judgement was that data saturation was reached, as nothing new was being added to the narrative as the analysis progressed. According to Saunders et al. (42), this type of information redundancy is a form of data saturation that is fairly easily identified with very prescribed research questions that are explored in niche populations. The interviews in this evaluation fit into this category.

## 3 Results

Three overarching themes and their subthemes are reported here. These broadly relate to motivations for and experiences of starting LAIB treatment, including physical and psychological impacts; experiences of treatment at the BPSS; treatment outcomes and the recovery process (**Figure 2**).

**Figure 2:**
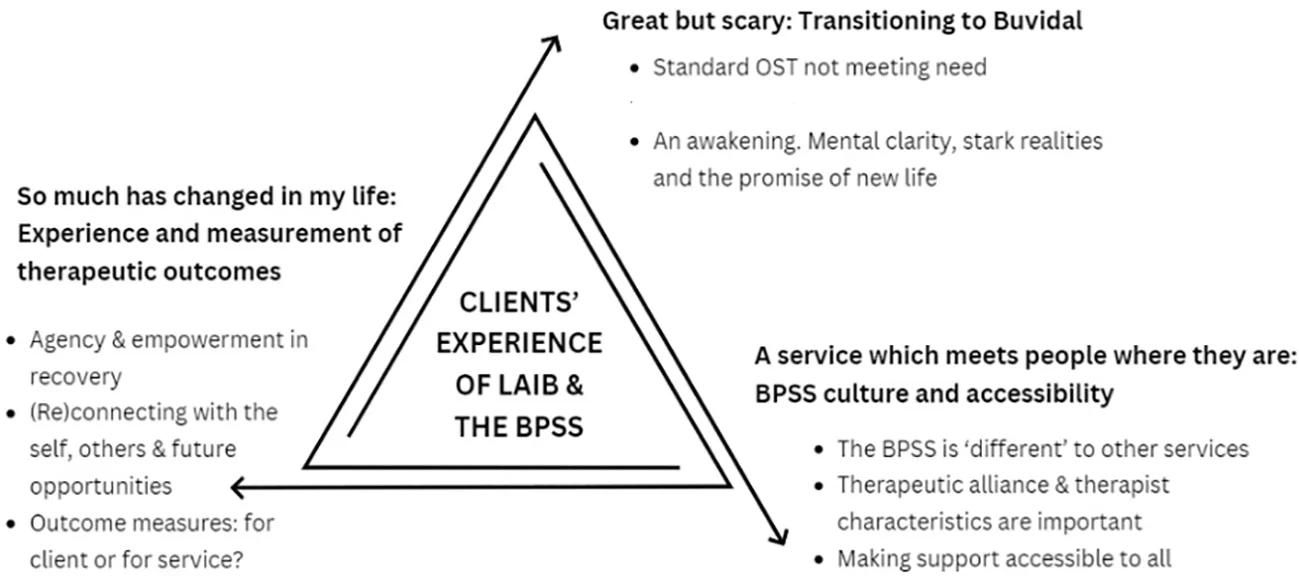
Themes.

### 3.1 Theme 1: “great but scary”: transitioning to Buvidal

This theme encapsulates the positive effects of LAIB comparative to standard OSTs, but also the unsettling nature of the transition.

#### 3.1.1 Standard OST not meeting needs

Clients’ transitions to Buvidal were typically driven by unmet personal and practical needs of traditional OSTs. The burdens associated with previous treatments impacted their lives in a variety of ways. These included the practicalities of *traveling* abroad with a controlled drug (P004), struggles with adherence (P005), an arduous daily treatment regimen (P003) and regular pharmacy attendance:

“*I was just fed up of constantly having to take tablets and constantly having to go to the pharmacy – another thing for me to have to be on top of*” (P001, L18-20).

P003 described how methadone and subutex had made them feel like they were “*still a junkie but just on a legal drug*” (L170-171).

Nor did standard treatments meet clients’ expectations of tolerability or efficacy, through contraindications with suicidal tendencies (P008) and a failure to inhibit problematic substance use (P002, P008). Buvidal was qualitatively different. Monthly administration freed up time and mental space (P001), and with it the opportunity to “start having a life again” (P003, L81). Its efficacy in promoting abstinence from chronic opioid use was illustrated by P008:

“*I’m coming up to a year off heroin. I started using heroin when I was 14 and now I’m 56, so that’s 42 years of using (…) I couldn’t have done it without Buvidal*” (L96-97).

Some also reported having witnessed the apparent efficacy of Buvidal *among* their peers, which had motivated their own transition. One client recalled his incredulity at the markedly high – and sustained – success rate:

“*… consider the other stuff. You see maybe one or two get clean every now and then, and half of them will be back again within a couple of months. But like the rate at which I was seeing them clear out and never see them again, I weren’t believing it (…) I was thinking like, my gosh … what’s going on, you know? This is a revolution like*” (P003, L136-142).

#### 3.1.2 An awakening: mental clarity, stark realities and the promise of new life

Spiritual language frequented clients’ descriptions of Buvidal. Its novelty, combined with little knowledge about how it works as a treatment, meant that some experienced the transition as a giving of oneself over to the unknown: “*once I handed my faith over to it … I was amazed*” (P003, L152); a commitment rewarded by the ‘*wonderous*’ (P008), ‘*life-giving*’ (P008), ‘*miracle drug*’ (P003, L71). Universally, there was a sense of an awakening, experienced by P008 as a momentous demarcation of life ‘before’ and ‘after’:

“*What I didn’t expect was one day I was a physical addict with the withdrawal symptoms, the aches and the pains, the sweats and everything that goes with it, and the following morning I woke up as well as I’ve woken up every morning since (…) not a single withdrawal symptom! Wow, can you imagine that after 40 years of putting the needle in your arm? You wake up every morning and you don’t have to do that*” (L16-23).

Although unanimous in their praise of Buvidal, especially in relation to its physical benefits, clients found that the psychological effects were not so benevolent.

Increased mental clarity meant an abrupt confrontation with many of the reasons underlying their OUD. No longer obscured by the effects of illicit substances or the lassitude associated with standard OSTs, psychological and trauma-related difficulties came into sharp focus:

“*At first, I didn’t like it because it was like just living a normal life and I found it hard to like deal with my emotions because everything comes back*” (P005, L15-17)

The importance of trauma-related psychological support was very evident from these client accounts. P008 expressed their fears had it not been available:

“*… that’s why it’s vital that you’ve got psychological support (…) Yes, [Buvidal has] worked, but all of a sudden (…) who’s there to help me, who’s there to catch me? You open a tin of worms but you don’t shut the lid on it again. I’m gonna be stuck and using possibly even harder drugs that I was using in the first place*” (P008, 32-40).

Concerns about relapse were echoed by P006, who described how their problematic substance use had been rooted in attempts “to dampen down feelings of suicide and depression” (L106-107). The urge to return to self-medicating *behavior*s was strong, demonstrating the BPSS’ role in supporting Buvidal adherence and maintaining abstinence. P008 was explicit in their belief that the difference between a successful Buvidal outcome (vs. relapse) was the psychological support they had received:

“*Some of my friends chose not to access the support and they ended up coming off the injection and going back onto the Class As*” (P008, L76-77).

For others, psychological support was needed to adjust to day-to-day life. Further, for some clients the new freedom from daily medication routines initially left them feeling somewhat at a loss:

“*… it sounds silly but it can be quite scary (…) I have been doing that over about 10 years or so and then boom! All of a sudden, it’s one injection a month and off you go and live your life. It’s great but it’s – it’s scary*” (P001, L208-209).

### 3.2 Theme 2: a service which meets people where they are: BPSS culture and accessibility

This theme represents the ways in which clients experienced the BPSS as ‘being there’ for them – in both a physical and psychological sense – at their point of need, wherever they were on their recovery journey. These reflections appear to validate the importance of the input of service users into the design of the service.

#### 3.2.1 The BPSS is ‘different’ to other services

Clients had many years of experience of other addiction, health and care services and drew *favorable* comparisons between the BPSS and these. This began with their initial contact, as the referral process and wait time exceeded expectations:

“*It’s an amazing service – it has been so quick (…) it’s probably been the quickest that I have experienced and I have experienced quite a lot of services*” (P001, L159-161).

Upon entry, clients noted how they were “*comfortable from the first time coming in*” (P004, L73), “*always felt cared for*” (P009, L137), and that it was a place of safety and trust (P007). Expressions of kindness and non-judgement were particularly valued; not solely located within the therapeutic relationship, rather they permeated interactions with the wider multidisciplinary team:

“*The staff – not just the therapist but the ladies in the office, the ladies over the road, the nurses, they’re all so supportive (…) everybody’s interested in what you’ve got to say*” (P008, L222-227)

Clients also reported that despite often having “*been in and out of different counsellors and things over the years*” (P003, L6-7), “*this was the first course of therapy that [they’d] actually completed*” (P008, L169). Many had previously struggled to engage due to a poor therapeutic alliance (P004), a culture not conducive to disclosure (P007), lack of therapeutic progress (P008), or being

“*on a lot of drugs - you can’t solve your problems in your head if you’re not compos mentis can you?*” (P003, L16-17).

P008 described how they had received multiple psychiatric diagnoses throughout their previous contacts with services telling them ‘what’ they were, which they felt stood in direct contrast to the person-*centered* approach of the BPSS whose focus was understanding ‘who’ they were:

“*I’m sure [previous services] like just putting labels on me: first traumatic stress, borderline personality, manic bipolar, there’s so many (…) people have stuck on me and said, you are this, you are that (…) Not to be judged (…) to have somebody not wanting to put labels on you, but to remove some of the labels, is a completely different experience (…) it is the opposite of what everything’s ever been*” (L273-285).

Praise for the BPSS’ positive culture radiated from the narratives, with P004 articulating a feeling shared by many clients:

“*everything was honestly just the best experience I had when it comes to mental health*” (L227-228).

#### 3.2.2 The therapeutic alliance and therapist characteristics are important

The consistency and continuity of seeing the same therapist for the duration of the specific tier was valued by clients, as it allowed them to “*build that relationship*” and “*build trust*” (P008, L177), particularly in the context of disclosing traumatic life events. The detrimental impact of this having not been the case in other services was palpable, as P008 recalled how they “*used to walk out and refuse to come back*”, such was their frustration and distress at having to relive *“[their] life story once and then go back next week and there’s a different person there, so [they’d have to] to tell them again*” (L170-172).

In contrast, at the BPSS “it didn’t feel like therapy sessions, it felt like talking to a friend” to whom they could disclose “absolutely anything” (L162).

For some, the client-practitioner alliance appeared to be a mediator of therapeutic change, and clients’ keen sense of loss upon their completion of sessions was testament to the profound connection they felt:

“*I don’t wanna get emotional, but like I only met the therapist eight times because I had eight sessions (…) When it finished, I was devastated. I’m still a bit devastated because (…) he just understood me*” (P004, L122-127).

#### 3.2.3 Making support accessible to all

The person-*centered* ethos of the BPSS was reflected in the way clients felt that the service was “*designed around [them], rather than [them] having to fit their life around it*” (P008, L255-257). Clients appreciated support having been brought ‘*to them*’ through at-home assessment (P008) (either online or by telephone), providing sessions at the LAIB dispensary (P005, P006) and by video call (P006), in addition to peer support (P008). During therapeutic work, clients valued the way in which their therapist “*tailored [sessions]*” (P004, 117) and made adaptions to accommodate their specific needs. Those with co-morbid health (P003, P002) or cognitive challenges (P005, P007) which impacted their ability to process or remember information, were supported with visual and audio resources.

### 3.3 Theme 3: so much has changed in my life: experience and therapeutic outcomes

This theme depicts the ways in which clients were supported to effect positive change in their lives.

#### 3.3.1 Agency and empowerment in recovery

Clients were receptive to furthering their psychological understanding of themselves “*because to beat your enemy, you need to know your enemy*” (P009, L119-12) and felt empowered to implement the skills they had learned in therapy to manage their symptoms. Techniques *focused* on emotional regulation and self-soothing appeared particularly useful:

“*When I wake up from really bad night terrors, I cry and stuff, and now I try to lay there and just breathe try and calm myself. [Therapist] told me how to do that properly and I do find that it does help*” (P006, L64-71).

Narratives revealed a robust comprehension of concepts such as the ‘*window of tolerance*’ (P004) and those derived from acceptance and commitment therapy (ACT), which clients had learned in their treatment sessions and proactively embedded into their everyday life:

“*[Imagine] leaves on the stream and an annoying thought comes along, put the thought on that leaf and watch it go down the river (…) there’s a couple of different analogies and I use them every day*” (P008, L192-195).

For those who had previously struggled to put their own needs first (P007) or “*do right by [them]self*” (P009, L460), completing therapy was their first defiant act of reclaiming agency over their lives. Armed with psychological tools, clients set about effecting positive change. P007 described how their newfound self-confidence and learning around interpersonal boundaries had enabled them to commit to leaving an unhealthy relationship. A sense of freedom and agency shared by P009, who no longer felt at the mercy of life happening ‘to’ them:

“*I’ve truly changed on a fundamental level. Like I’m free, I’m free. I’m free to control myself and my emotions and my actions. I’ve got my freedom back. I’m not a prisoner in my own body anymore (…) I’ve always thought throughout my whole life, like I was looking from outside, looking through the window. And I never understood what it was, and it was because I wasn’t in control. I was literally a bystander to myself*” (P009, L436-445).

#### 3.3.2 (Re)connecting with the self, others and seeing future opportunities

Wide-ranging clinically meaningful benefits were reported by clients. Woven throughout was a profound sense of (re)connection: with one’s own humanity, a new identity, family, wider society, and with a hope-filled future once thought to be unattainable. The narratives of P003 and P009 illustrated journeys of reconnecting with the self. Both painted stark pictures of themselves as being sub-human in some way, prior to LAIB and the BPSS. P003 described how low self-worth, social isolation and poor living conditions had left him feeling “*like a rat*” (L258), while P009 saw their past self as “*a dead man walking*” (L415); profound psychological distress having robbed him of his humanity:

“*That thing that makes you a human being – that light – it had been snuffed out for years. I was venomous. I could have just walked around and punch people in the face and it probably would have felt good (…) that’s how hateful, you know? It was all consuming. It was awful. Because I honestly was in agony*” (L26-32).

Having lost years of formative self-discovery to trauma and addiction, both were reclaiming their “*sense of identity*” (P009, L366). P009 articulated how engaging in trauma work and EMDR had released him from the effects of PTSD which he had experienced since childhood and so was “*literally getting to know [him]self for the first time*” (L393). Others reconnected with the self through the values of self-compassion and self-acceptance; stopping self-harming (P004) and working to overcome the shame associated with past negative behaviors (P005).

P008 described how they had re-built relationships with their son and grandchildren.

Clients also felt they had regained their society membership; through activities (P003), housing (P008) and education (P003, P004, P008). P004 had enrolled onto a counselling course, so inspired were they by the support received from the BPSS:

“*I’m excited because I’ve always wanted to help people since I found myself in this situation (…) hopefully one day, I’ll be able to do the same and help people (…) like the therapist helped me*” (L239-243).

#### 3.3.3 Outcome measures: for client or for service?

As a new psychology-driven service, the BPSS was cognizant of the need to understand its effectiveness and efficacy through a range of outcome measures. Some clients viewed these as a ‘*necessary evil*’ of the service that detracted from therapy time (P001, P007), but others as a personal tool to evaluate their progress (P008) and as objective representation of their internal sense of therapeutic change:

“*It’s all well and good feeling like you’ve changed, but when you see it on paper and you see your answers previously to how you’re answering now. You can look back on where you started and where you’ve arrived at, it’s external confirmation (…) I could see the old me and the new me on paper quite literally in front of my eyes*” (P009, L216-225).

There was, however, a shared positive view of the provision of a follow-up report, which summarized individual journeys with the BPSS:

“*… everything they helped with and my story, and that was really nice to have. That’s nice to look back on*” (P004, L217-220).

## 4 Discussion

This evaluation of the BPSS offers novel insights into how an integrated, trauma-informed psychological support model can address the complex needs of individuals with OUD who are receiving LAIB. Clients consistently endorsed the BPSS as a markedly different and more positive experience compared to previous services. This finding aligns with literature underscoring the importance of therapeutic alliance, continuity of care, and a non-judgmental, person-centered approach in improving engagement and outcomes for individuals with SUD and comorbid mental health conditions (43, 44). These findings extend previous LAIB research by showing that the benefits extend beyond reduced withdrawal and improved convenience, and into the social and emotional domain of recovery. Prior studies have highlighted improved satisfaction, reduced stigma, and better quality of life with LAIB (45, 46), but less was known about what happens when improved pharmacological stability brings underlying distress into sharper focus.

The present data suggest that transitioning to Buvidal was both transformative and challenging for clients making psychological support a central rather than optional component of care for these clients. The pharmacological benefits, including the reduction in withdrawal symptoms and opioid cravings, enabled a powerful sense of awakening and regained agency in recovery. However, the increased mental clarity also exposed clients to the full impact of unresolved trauma and psychological symptoms that had been masked by illicit opioid use or the sedative side effects of older OSTs (47). This supports recent literature suggesting that LAIB can be enabling in important social and emotional ways, while also bringing new vulnerabilities into view as people adjust to life with greater clarity and reduced dependence on daily treatment routines (48). This paradoxical effect of pharmacological stabilization precipitating psychological distress underscores the importance of embedding comprehensive trauma-focused psychological care alongside pharmacological treatment to optimize recovery outcomes for this population.

These findings illustrate the risks of relapse attributable to untreated trauma and mental health conditions once opioid dependence is physically managed (49). This is consistent with Fish et al. (46), who found that LAIB was experienced as “life changing” in community settings, but only when accompanied by the wraparound support offered by some services, including continuity, accessible information, and multi-service collaboration. Several clients emphasized that without timely access to psychological support, the resurgence of emotional pain and trauma memories could lead to a return to substance use. The tiered intervention approach used by BPSS appears to effectively match client needs with appropriate intensity of care, ranging from brief trauma-informed sessions to intensive trauma-processing therapies.

Clients’ descriptions of “reconnecting” with self, others, and future possibilities reveal profound psychosocial gains facilitated by the BPSS. These accounts align with Herman’s (38) trauma recovery model, particularly the restoration of safety, agency, and connection. The emergence of self-compassion appeared to support emotional regulation, while increased empowerment enabled clients to challenge maladaptive relational dynamics and exercise greater choice in their recovery. Furthermore, accounts of lost identities being restored and social networks being rebuilt articulate recovery as a rehumanizing process grounded in reconnection with self, others, and future possibilities. This process challenges the stigma, disempowerment, and social isolation pervasive among people with SUDs (50, 51).

This evaluation’s strengths are capturing subjective lived experience and the focus on a unique LAIB-linked psychological service. However, limitations must be acknowledged. The sample may reflect a degree of selection bias, as participation required both engagement with BPSS and involvement in interviews, possibly limiting inclusion of those with poorer outcomes or disengagement. The sample was small and drawn through opportunity sampling from one service context, which may limit transferability. Participants who agreed to take part may also have been more engaged with BPSS or more likely to report positive experiences than those who declined or disengaged. Future research should seek to document the experiences of these service users as well. In addition, the use of BPSS staff as interviewers may have contributed to response bias, with participants potentially moderating their accounts. The findings reflect participants’ accounts of perceived impact and should not be interpreted as demonstrating effectiveness in an outcome-evaluative sense.

The BPSS model offers a replicable framework incorporating trauma-informed principles and flexible care delivery modalities that promote accessibility and client-centeredness. Integrated, tiered trauma-focused psychological interventions alongside LAIB can better address the complexity of co-occurring PTSD, psychological distress, and substance use, with potential to improve retention, reduce relapse, and promote sustained recovery.

In conclusion, the BPSS pilot highlights the potential value of integrated trauma-focused psychological support in the care of individuals receiving LAIB for OUD who experience mental health problems. Through a combination of pharmacological stabilization and trauma-informed therapeutic engagement, the BPSS appeared to provide a context in which some clients could begin to address underlying trauma, reducing psychological distress, improving functioning, and fostering transformative recovery. These findings advocate for systemic integration of psychological services within LAIB treatment frameworks to optimize outcomes in this vulnerable population.

## 5 Dedication

We dedicate this paper to Professor Jan Melichar, who sadly and unexpectedly died on August 31st 2025. His vision, commitment, and tireless efforts were pivotal in the rollout of LAIB across Wales and the establishment of the BPSS. His legacy endures in the transformation of opioid dependence treatment and the countless lives saved.

## Data Availability

The datasets presented in this article are not readily available because the data are not publicly available due to privacy or ethical restrictions. Requests to access the datasets should be directed to Lucie James, lucie.james@wales.nhs.uk.
